# A meta-analysis of the Zilongjin tablets for non-small cell lung cancer and its network pharmacology of action against NSCLC and COVID-19

**DOI:** 10.3389/fmed.2023.1080121

**Published:** 2023-08-01

**Authors:** Wuxia Yang, Yichao Zhang, Jing Gao, Pengcheng Hu, Yanjie Yang, Xiaoqing Xu

**Affiliations:** ^1^The Graduate School, Tianjin Medical University, Tianjin, China; ^2^The Graduate School, Qinghai University, Xining, China; ^3^Clinical Laboratory, First Teaching Hospital of Tianjin University of Traditional Chinese Medicine, Tianjin, China; ^4^National Clinical Research Center for Chinese Medicine Acupuncture and Moxibustion, Tianjin, China; ^5^Department of Ophthalmology, The Second Affiliated Hospital of Chongqing Medical University, Chongqing, China; ^6^Shunde Hospital of Southern Medical University, Foshan, China

**Keywords:** Zilongjin tablets, non-small cell lung cancer (NSCLC), COVID-19, traditional Chinese medicine, meta-analysis, network pharmacology

## Abstract

**Objective:**

To objectively evaluate the efficacy of the Zilongjin tablets in non-small cell lung cancer (NSCLC) and to explore its potential mechanism of action against NSCLC and COVID-19 based on network pharmacology.

**Methods:**

The database was searched for randomized controlled trials (RCTs) of the Zilongjin tablets for NSCLC published up to 22 August 2022. The quality of included trials was assessed using Cochrane standard guidelines, and a meta-analysis was performed using Rev Man 5.3. Gene targets for intersections of NSCLC and COVID-19 (the NC) and drugs were obtained from the TCMSP database, HERB database, GeneCards database, and the NCBI database for network pharmacology research.

**Results:**

Meta-analysis included 14 articles with 2,430 patients. The meta-analysis showed that the Zilongjin tablets combined with conventional chemotherapy were significantly more effective than chemotherapy alone in the treatment of NSCLC. A total of 29 drug-disease intersecting targets were identified in the network pharmacology. The “ingredient-target-pathway” diagram component-target-pathway network contained 119 nodes and 429 edges, with the majority of targets associated with inflammatory responses.

**Conclusion:**

The efficacy and quality of life of the Zilongjin tablets combined conventional chemotherapy for NSCLC were significantly better than chemotherapy alone, alleviating various adverse effects. At the same time, the Zilongjin tablets may modulate the inflammatory response to alleviate NSCLC and COVID-19.

## 1. Introduction

Lung cancer has a high incidence and mortality rate, with approximately 1.8 million new cases and 1.6 million deaths per year, and the 5-year survival rate of lung cancer ranges from 4 to 17%. Moreover, it is the leading cause of cancer-related death among men; and the second leading cause of cancer-related death among women worldwide (1, 2). Non-small cell lung cancer (NSCLC) accounts for about 84% of all types of lung cancer and has a poor prognosis, especially in advanced stages (3, 4). Treatment of lung cancer includes surgery, chemotherapy, radiotherapy, molecular targeted therapy, and immunotherapy (5). Currently, chemotherapy is still the first choice for lung cancer treatment, however, the more severe the disease progression, the less tolerant patients are to chemotherapy, due to the painful disease symptoms and severe adverse effects caused by chemotherapy.

At the time of the COVID-19 pandemic, a large number of lung cancer patients face difficulty in admission to a hospital for treatment, and their risk of COVID-19 infection is 2.31 times higher than that of the general population, due to immunosuppression and respiratory infections (6–9). When lung cancer patients are infected, they have a higher risk of disease progression, so COVID-19 combined with NSCLC leads to a higher mortality rate and a worse prognosis than the general population. Currently, the National Institute for Health and Care Excellence (NICE) has published a clinical guideline for standard anticancer treatment for cancer patients with COVID-19 while the available studies do not appear specific values for the effect of COVID-19 treatment (10–12, 14). Additionally, patients may experience lung damage symptoms for a while after the COVID-19 fever goes away, including fatigue, shortness of breath, drowsiness, dizziness, loss of appetite, shortness of breath, sweating, coughing, and chest pain—the same side effects as those brought on by chemotherapy for NSCLC. The pathogenesis of both conditions is characterized by qi and blood insufficiency, unexhausted residual heat, and a connection between phlegm and blood stasis. Therefore, the similarity of symptoms and pathogenesis may suggest that they are mechanistically related.

A large number of clinical studies have shown that traditional Chinese medicine combined with chemotherapy can improve not only the anti-tumor effect but also enhance the immune function of the body (11). The Zilongjin tablet is a tonifying formula that has the advantageous effects of regulating qi, nourishing blood, clearing heat, detoxifying, and removing phlegm, having an anti-tumor function, improving the quality of life of tumor patients, reducing the adverse effects of chemotherapy, etc. Its ingredients mainly include the following eight herbs, Astragali Radix (AR, Huangqi in Chinese), Angelicae Sinensis Radix (ASR, Danggui in Chinese), Herba Solani Lyrati (HSL, Baimaoteng in Chinese), Solanum Nigrum Linn (SNL, Longkui in Chinese), Salviae liguliobae Radix (SLR, Danshen in Chinese), Scutellariae Barbatae Herba (SBH, Banzhilian in Chinese), Curcumae Radix (CR, Yujin in Chinese), and Duchesnea indica (DI, Shemei in Chineses) (12). In order to assess the efficacy of the Zilongjin tablets on NSCLC more objectively and provide evidence-based medical evidence, we comprehensively evaluated the efficacy of the Zilongjin tablets for NSCLC by meta-analysis. Besides, the study is a basis for follow-up studies to analyze the potential mechanism of the Zilongjin tablets on the intersection gene of NSCLC and COVID-19 (the NC) based on network pharmacology.

## 2. Method

### 2.1. Database and search strategy

The following electronic databases were searched to identify eligible trials: Chinese National Knowledge Infrastructure (CNKI), Wan-fang Database, China BioMedical Literature (CBM), MEDLINE (PubMed), EMBASE, Google Scholar, World Health Organization International Clinical Trials Registry (http://www.who.int/ictrp/en/), and US National Institutes of Health Ongoing Trials Register (ClinicalTrials.gov). Meanwhile, eligible trials were retrieved from the initial date until August 22, 2022. The following keywords and phrases, and their abbreviations or derivatives, were used separately or in combination: “the Zilongjin tablets” and “non-small cell lung cancer” or “NSCLC” and “RCT”. The search terms were adapted to different databases with a reliable search strategy developed by the Cochrane Collaboration (13). Finally, two reviewers managed and analyzed all studies by Zotero independently.

### 2.2. Study selection

#### 2.2.1. Inclusion criteria: type of studies

All RCTs, published before August 22, 2022, were considered regardless of blinding.

#### 2.2.2. Type of participants

Any patients with NSCLC of any ethnicity, gender, profession, and age were included.

#### 2.2.3. Type of interventions

The experimental group was the Zilongjin tablets combined with a chemotherapy drug, and the chemotherapy drug combined with and without a placebo group was the control group.

### 2.3. Outcomes

The primary outcome includes: Objective response rate (ORR); disease control rate (DCR); Quality of life; and Adverse reactions.

### 2.4. Studies selection and data extraction

Two reviewers independently screened the titles and abstracts of searching results against pre-specified inclusion criteria to identify potential relevance. Disagreements were resolved by consensus. The third reviewer judged all articles included. Two reviewers systematically extracted data regarding study design, participant characteristics, interventions, and outcomes independently. Similarly, discrepancies were resolved by discussion between the two reviewers and consultation with the third reviewer.

#### 2.4.1. Types of study designs to be included

We included randomized controlled trials (RCTs) irrespective of blinding or language. We excluded quasi-randomized trials.

#### 2.4.2. Data extraction and management

Two reviewers independently extracted data, including details of the study population, intervention, and outcome, using a predesigned data extraction form. The following data were extracted: gross trial characteristics (title, authors, and year); baseline of the patient and disease data (gender, age, and sample size); interventions ways; and outcomes (outcome measures and the rate of adverse events). We resolved differences in data extraction by consensus or a third party. One reviewer entered data into Cochrane software Review Manager 5.3 (RevMan5.3) (14), and checked the data to reduce data entry errors.

### 2.5. Quality assessment

The risk of bias evaluation tool proposed by the Cochrane Collaboration was utilized to address the following seven domains: random sequence generation, allocation concealment, blinding of participants and personnel, blinding of outcome assessment, incomplete outcome data, selective outcome reporting, and “other bias” (15). The risk of bias for each item was summarized into three categories: low, unclear, and high. The first reviewer performed the quality assessment with supervision from the other two reviewers.

### 2.6. Data synthesis and measures of treatment effect

The data were analyzed using Review Manager 5.3 software (14). Risk ratios (RRs) were calculated using the Mantel–Haenszel method for dichotomous outcomes, and weighted mean differences (MDs) were calculated using the inverse variance method for continuous variables. We used I^2^ statistics to assess the heterogeneity. A fixed-effect (FE) model was used if there was no significant heterogeneity in the data (I^2^ < 50%), and a random-effect (RE) model was used if significant heterogeneity was present (I^2^ > 50%) (16).

### 2.7. Screening of drug components

The names of all the ingredients of Zilongjin Tablets were retrieved through the TCMSP database (https://tcmspw.com/tcmsp.php), HERB database (http://herb.ac.cn/). Furthermore, all the drug components were collated and retrieved in the PubChem database (https://pubchem.ncbi.nlm.nih.gov/) to obtain the CID numbers, Smiles numbers, and canonical molecular formulas of the active ingredients by Python. The detected drug compounds were imported into the STITCH database (http://stitch.embl.de/cgi/input.pl) to obtain the gene targets interacting with the active ingredient of the Zilongjin tablets, selecting “Homo sapiens”, and screening the targets with a combined score >0.95. We also obtained the gene targets from the HERB database (*P* < 0.05). Then, we collated all gene targets and screened out duplicate items.

### 2.8. Retrieval of disease gene targets

The disease targets about the NSCLC and COVID-19 were retrieved from the GeneCards database (https://www.genecards.org) and the NCBI database (https://www.ncbi.nlm.nih.gov) respectively. Additionally, we de-duplicated them with scores >10 in the GeneCards database. Bioinformatics & Evolutionary Genomics (http://bioinformatics.psb.ugent.be/webtools/Venn/) was used to intersect the genes of NSCLC and COVID-19, and the two disease intersections are referred to as the NC.

### 2.9. Screening of the gene targets of the drug-disease

Using the Bioinformatics & Evolutionary Genomics website, the gene targets of the Zilongjin tablets were intersected with the NC disease targets to obtain the targets of the Zilongjin tablets for both diseases. The 29 targets obtained by screening were imported into the STRING database (http://string-db.org) for the protein-protein interaction (PPI) network. We selected the species as “Homo sapiens”, and the protein interaction score as “high confidence > 0.9”, hiding the unlinked nodes, and kept the rest of the parameters unchanged to obtain the PPI network map. Furthermore, we obtained the interactions information file “string_interactions.tsv” from the String database, imported the data into Cytoscape 3.8.2 software to restring the topology of analysis results by “Network Analyzer” function, and made the PPI network map according to the degree value.

### 2.10. Enrichment analysis

The intersecting targets of the Zilongjin tablets drug components and the NC were imported into The Database for Annotation, Visualization, and Integrated Discovery (DAVID, https://david.ncifcrf.gov/home.jsp) for Gene Ontology (GO) analysis, including biological process (BP), cellular component (CC), and molecular function (MF), and Kyoto Encyclopedia of Genes and Genomes (KEGG). The results were visualized and analyzed using a microbiology letter (http://www.bioinformatics.com.cn/login/) (17, 18). Meanwhile, we performed a visual topological analysis of the KEGG pathway by applying Cytoscape 3.8.2.

### 2.11. “Component-target-pathway” networks of the Zilongjin tablets and the NC

We obtained the drug components corresponding to the intersecting targets from the HERB database, and afterward, we docked it with the specific active ingredients of the Zilongjin tablets. Cytoscape 3.8.2 software was used to construct the active ingredient-the NC target network of the Zilongjin tablets, and “merge” and “group attributes layout” were used to cluster the active ingredients. The node size was described by “degree.”

## 3. Results

### 3.1. Literature search process

**Figure 1:**
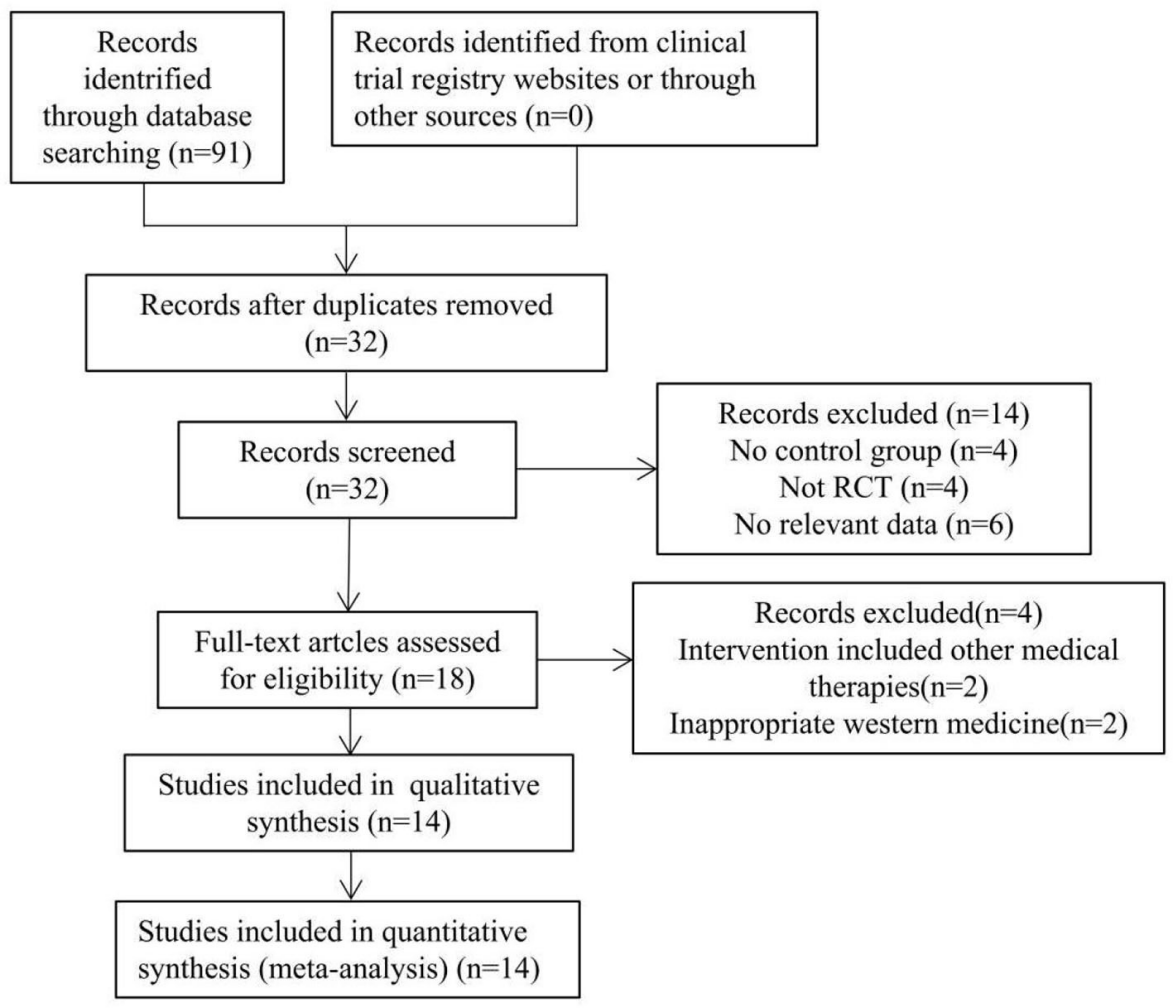
Flow chart of study search and selection process.

The flowchart of the study search results is shown in Figure 1. According to the inclusion and exclusion criteria, by reading the title, abstract, and full text of the literature, and excluding the repeated studies, cell and animal experiments, reviews and literature with inconsistent research contents, 14 RCTs that met the criteria was finally included (19–32), all of which were Chinese kinds of literature. The basic information of the included studies is shown in Table 1.

**Table 1:** Characteristics of the included trials.

**References**	**Number (T/C)**	**Gender (M/F)**	**Age (Y)**		**Intervention (T/C)**		**Treatment Duration (D)**	**Outcomes**
Chen et al. (19)	25/23	24/24	NE	NE	The Zilongjin tablets + chemotherapy	Chemotherapy	42	
Ding et al. (20)	20/22	30/12	NE	NE	The Zilongjin tablets + chemotherapy	Chemotherapy	42	
Li and Ma (21)	39/39	37/41	63 ± 8	60 ± 5	The Zilongjin tablets + chemotherapy	Chemotherapy	42	
Li et al. (22)	40/40	48/32	60.4 ± 2.8	60.4 ± 2.8	The Zilongjin tablets + chemotherapy	Chemotherapy	42	
Liu (23)	37/33	40/30	58 ± 5.7	56 ± 6.2	The Zilongjin tablets + chemotherapy	Chemotherapy	42	
Ma et al. (24)	39/39	NE	NE	NE	The Zilongjin tablets + chemotherapy	Chemotherapy	14	
Sun et al. (25)	64/64	85/43	56.5 ± 5.6	55.3 ± 4.9	The Zilongjin tablets + chemotherapy	Chemotherapy	42	
Shang (26)	30/30	35/25	66.8 ± 1.2	65.8 ± 1.8	The Zilongjin tablets + chemotherapy	Chemotherapy	84	
Shen et al. (27)	43/43	55/41	57.2 ± 7.3	56.8 ± 7.5	The Zilongjin tablets + chemotherapy	Chemotherapy	84	
Wang and Tan (28)	32/31	37/26	NE	NE	The Zilongjin tablets + chemotherapy	Chemotherapy	36	
Wang et al. (29)	40/38	48/30	NE	NE	The Zilongjin tablets + chemotherapy	Chemotherapy	42	
Yu and Jiang (30)	40/40	44/36	61.8 ± 7.7	60.4 ± 7.9	The Zilongjin tablets + chemotherapy	Chemotherapy	28	
Yang et al. (31)	62/62	82/42	61.3 ± 13.4	61.3 ± 13.4	The Zilongjin tablets +Chemotherapy	Chemotherapy	42	
Yang et al. (32)	25/24	29/20	48.65 ± 16.12	48.30 ± 16.24	The Zilongjin tablets + chemotherapy	Chemotherapy	42	

T, treatment group; C, control group; NE, No Explanation. Observation index: Objective remission rate (ORR), Disease control rate (DCR), Quality of life improvement rate, Adverse reaction.

### 3.2. Characteristics of the included trials

The characteristics of the included 14 trials are summarized in Table 1. A total of 1,064 participants were involved, of which 536 and 528 were in the treatment and control groups.

### 3.3. Quality assessment of included studies

**Figure 2:**
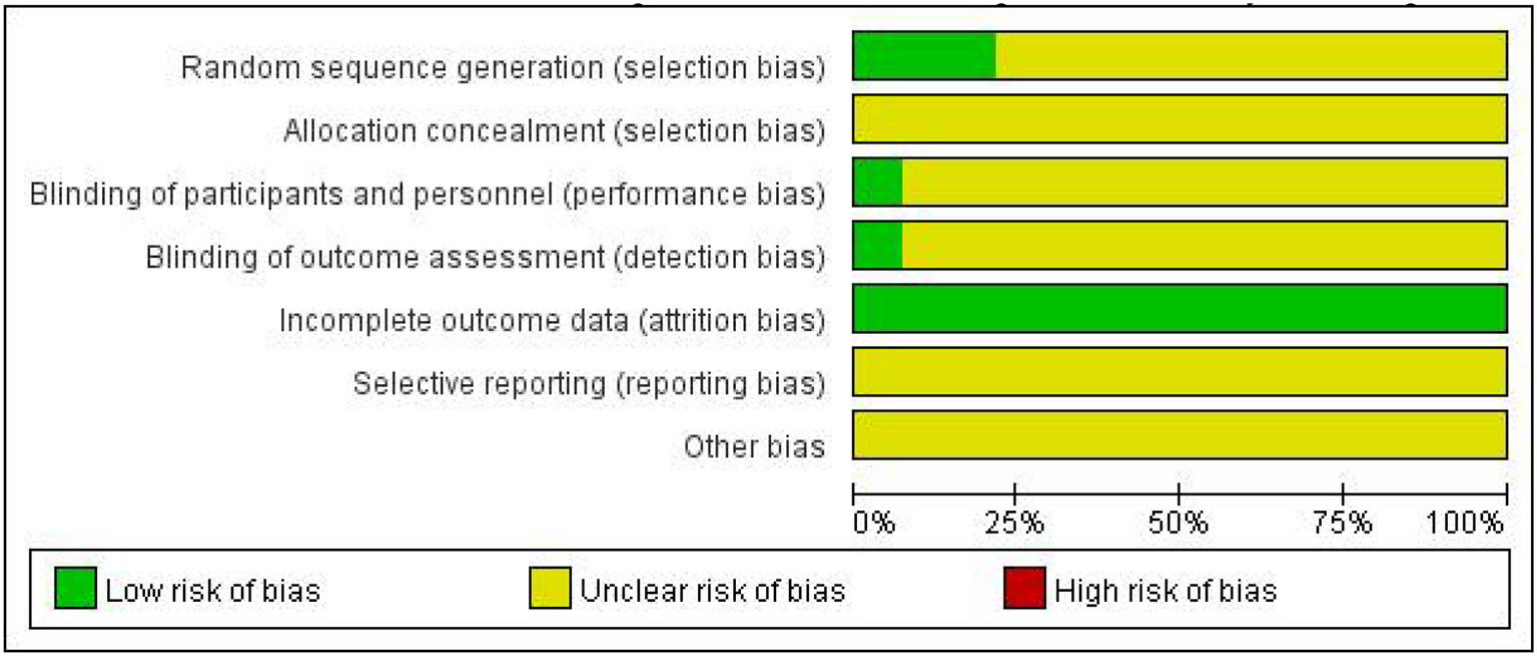
The Cochrane risk of bias.

**Figure 3:**
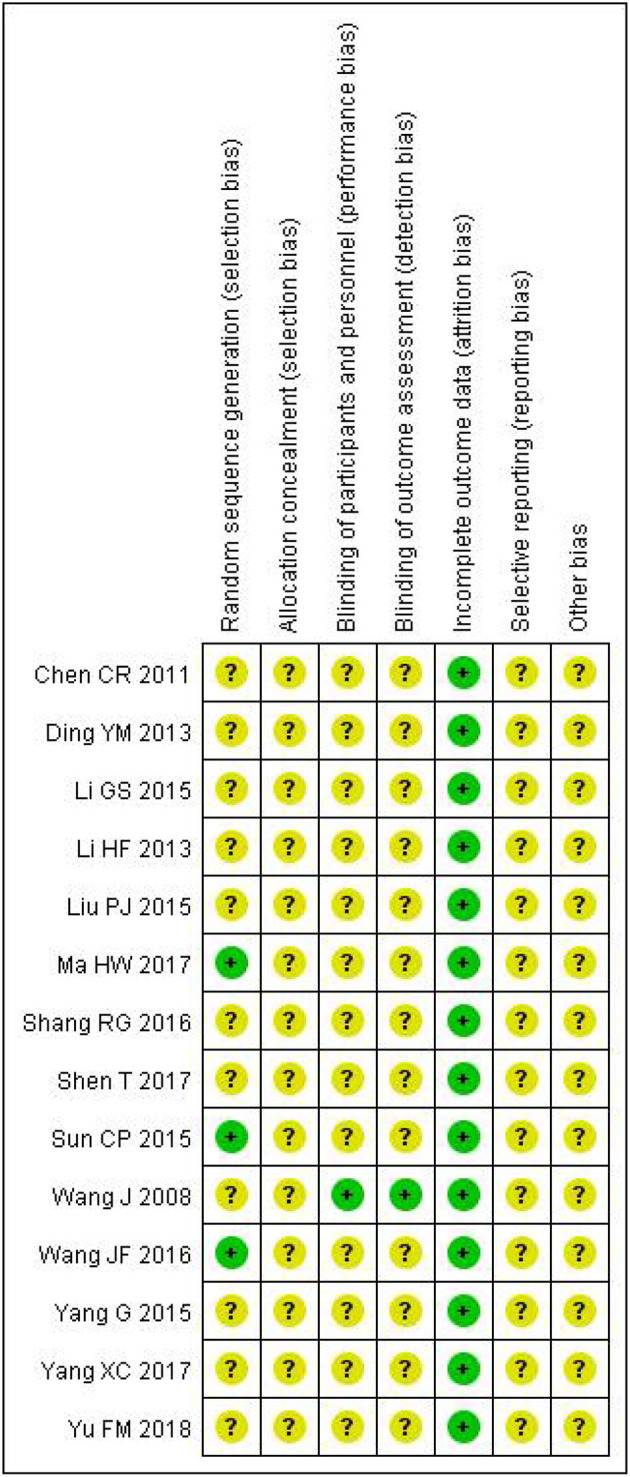
Risk of bias summary. The symbol “+”, low risk; the symbol “?”, unclear; the symbol “-”, high risk.

As shown in Figures 2, 3, unclear risk of bias is in the majority as minimal information was available in many studies to permit a judgment of whether the risk of bias existed. All trials claimed that they were RCTs and described inclusion/exclusion criteria as well as grouping methods. Of the 14 RCTs, 3 three studies specified that they used a random number table to realize the randomization, while the others did not report the randomization procedures. Only 1 trial reported double-blind.

### 3.4. Objective response rate

**Figure 4:**
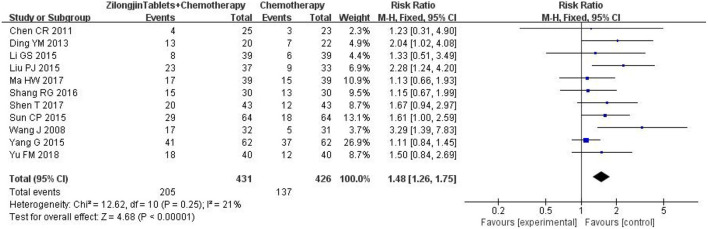
The ORR of the Zilongjin tablets combined with chemotherapy in the treatment of NSCLC.

A total of 11 studies with 857 subjects were included. There was no statistical heterogeneity among the 11 studies [Chi^2^ = 12.62, DF = 10 (*P* = 0.25); I^2^ = 21%] (Figure 4). Meta-analysis using the fixed-effect model showed that the improvement ORR of the Zilongjin tablets combined with chemotherapy in the treatment of NSCLC was significantly better than that of chemotherapy alone [OR = 1.48, 95%CI (1.26, 1.75), OR combined hypothesis test Z = 4.68, *P* < 0.0001].

### 3.5. Disease control rate

**Figure 5:**
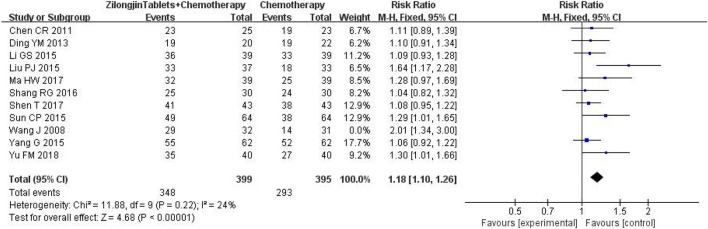
The DCR of the Zilongjin tablets combined with chemotherapy in the treatment of NSCLC.

A total of 11 studies with 857 subjects were included by heterogeneity test, I^2^ = 54 > 50%, indicating heterogeneity among studies. The sensitivity analysis of 11 kinds of literature showed that the study of Wang J greatly impacted heterogeneity. After removing this research, the heterogeneity test was conducted again, and the results showed that there was no heterogeneity in the remaining literatures (I^2^ = 24%) (Figure 5). Meta-analysis using the fixed-effect model showed that the improvement DCR of the Zilongjin tablets combined with chemotherapy in the treatment of NSCLC was significantly better than that of chemotherapy alone [OR = 1.18, 95%CI (1.10, 1.26) OR combined hypothesis test Z = 4.68, *P* < 0.0001].

### 3.6. Quality of life improvement rate

**Figure 6:**
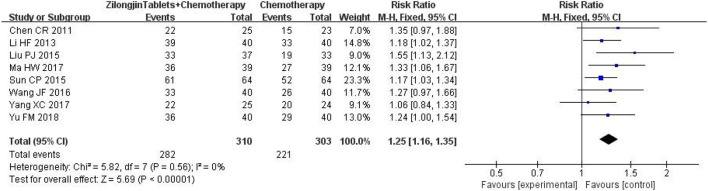
Quality of life improvement rate.

A total of 8 studies with 313 subjects were included. There was no statistical heterogeneity among the 8 studies [Chi^2^= 5.82, DF = 7 (*P* = 0.56); I^2^ = 0%] (Figure 6). Meta-analysis using the fixed-effect model showed that the improved quality of life of the Zilongjin tablets combined with chemotherapy in the treatment of NSCLC was significantly better than that of chemotherapy alone [OR = 1.25, 95%CI (1.16, 1.35), OR combined hypothesis test Z = 5.69, *P* < 0.0001].

### 3.7. The adverse event

**Figure 7:**
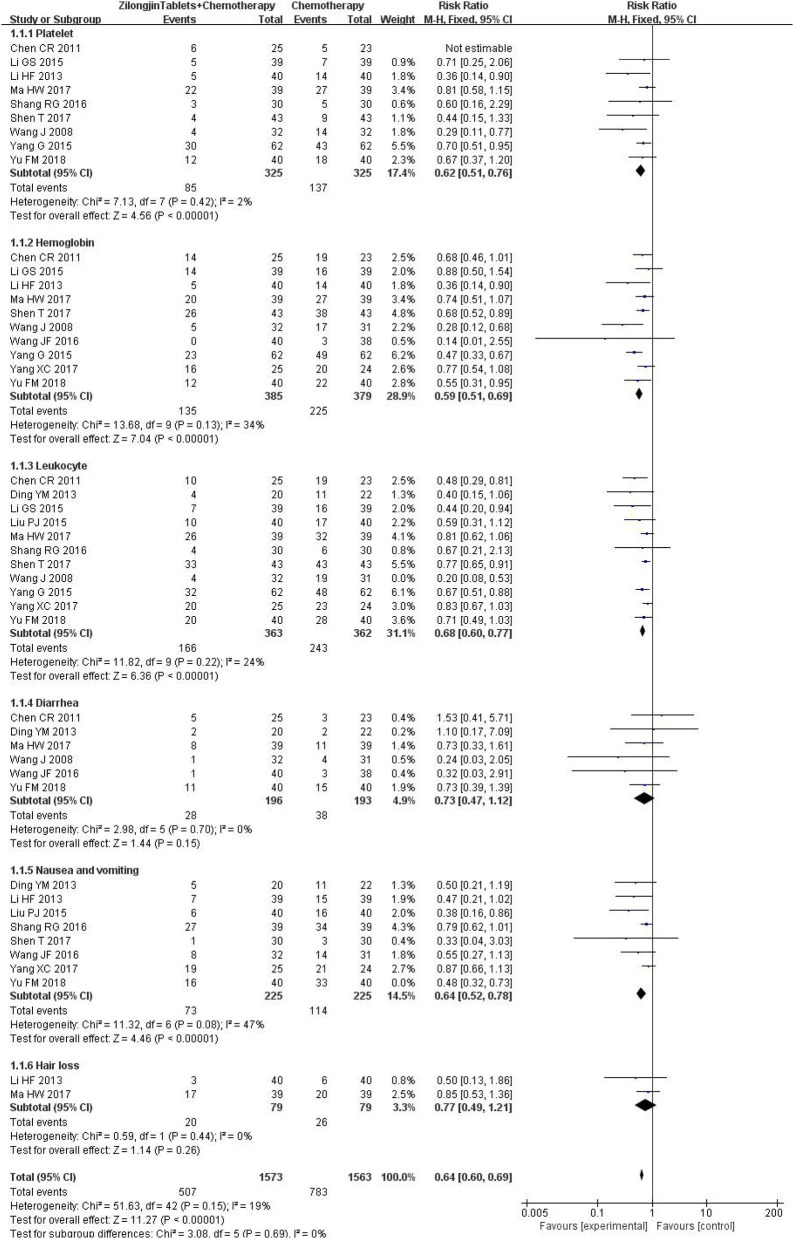
The adverse event.

Platelet counts were reported in 9 studies (650 cases), and there was no statistical heterogeneity [Chi^2^ = 7.13, DF =7 (*P* = 0.42); I^2^ = 2%). The fixed effect model was used for meta-analysis (Figure 7). The results of the meta-analysis showed that the platelet count of the experimental group was significantly higher than that of the control group, and the difference was statistically significant [RR = 0.62, 95%CI (0.51, 0.76), Z = 4.56, *P* < 0.00001].

Hemoglobin was reported in 11 studies (849 cases), and there was no statistical heterogeneity [Chi^2^ = 13.68, DF = 9 (*P* = 0.13); I^2^ = 34%]. The fixed effect model was used for meta-analysis. The results of the meta-analysis showed that the hemoglobin of the experimental group was significantly higher than that of the control group, and the difference was statistically significant [RR = 0.59, 95%CI (0.51, 0.69), Z = 7.04, *P* < 0.000 01].

Leukocytes were reported in 11 studies (725 cases), and there was no statistical heterogeneity [Chi^2^ = 11.82, DF = 9 (*P* = 0.22); I^2^ = 24%]. The fixed effect model was used for meta-analysis. The results of the meta-analysis showed that the leukocyte of the experimental group was significantly higher than that of the control group, and the difference was statistically significant [RR = 0.68, 95%CI (0.60, 0.77), Z = 6.36, *P* < 0.00001].

Diarrhea was reported in 6 studies (389 cases), and there was no statistical heterogeneity [Chi^2^ = 2.98, DF = 5 (*P* = 0.70); I^2^ = 0%]. The fixed effect model was used for meta-analysis. The results of the meta-analysis showed that there was no significant difference in diarrhea between the experimental group and the control group [RR = 0.73, 95%CI (0.47,1.12), Z = 1.44, *P* = 0.15 > 0.05].

Nausea and vomiting were reported in 8 studies (450 cases) [Chi^2^ = 11.32, DF = 6 (*P* = 0.08); I^2^ = 47%]. The fixed effect model was used for meta-analysis. The results of the meta-analysis showed that nausea and vomiting of the experimental group were significantly lower than that of the control group, and the difference was statistically significant [RR = 0.64, 95%CI (0.52,0.78), Z = 4.46, *P* < 0.00001].

Hair loss was reported in 2 studies (158 cases), and there was no statistical heterogeneity [Chi^2^ = 0.59, DF = 1 (*P* = 0.44); I^2^ = 0%]. The fixed effect model was used for meta-analysis. The results of the meta-analysis showed that there was no significant difference in hair loss between the experimental group and the control group [RR = 0.77, 95%CI (0.49,1.21), Z = 1.14, *P* < 0.26 > 0.05].

### 3.8. Drug' number in network pharmacology database

AR (Herb id, HERB002560; TCMSP id, 187). ASR (Herb id, HERB001210; TCMSP id, 95). HSL (Herb id, HERB000246; TCMSP id, 16). SNL (Herb id, HERB003482; TCMSP id, 252; SymMap id, SMHB00252). SLR (Herb id, HERB001193; TCMSP id, 93; SymMap id, SMHB00093). SBH (Herb id, HERB000338; TCMSP id, 36; SymMap id: SMHB00036). CR (Herb id; HERB006718; TCMSP id, 469; SymMap id: SMHB00469). DI (Herb id, HERB004977).

### 3.9. Results of drug component

A total of 149 active ingredients of AR, 276 of ASR, 104 of HSL, 94 of SNL, 253 of SLR, 113 of SBH, 279 of CR, and 21 of DI. Additionally, a total of 787 after de-weighting. Then, we obtained the stitch-PPI networks of the drugs (Table 2).

**Table 2:** Stitch-PPI networks of the drugs.

**Drug**	**Number of nodes**	**Number of edges**	**Average node degree**	**Clustering coefficient**	**Expected number of edges**	**PPI enrichment *p*-value**
AR	20	31	3.1	0.74	20	0.0119
ASR	19	38	4	0.875	24	0.00482
HSL	23	66	5.74	0.846	45	0.00162
SNL	21	38	3.62	0.76	27	0.0246
SLR	18	45	5	0.734	27	0.000822
SBH	5	10	4	1	4	0.00538
CR	7	15	4.29	1	5	0.000555
DI	20	31	3.1	0.755	20	0.0158

AR, Astragali Radix (Huangqi in Chinese); ASR, Angelicae Sinensis Radix (Danggui in Chinese); HSL, Herba Solani Lyrati (Baimaoteng in Chinese); SNL, Solanum Nigrum Linn (Longkui in Chinese); SLR, Salviae liguliobae Radix (Danshen in Chinese); SBH, Scutellariae Barbatae Herba (Banzhilian in Chinese); CR, Curcumae Radix (Yujin in Chinese); DI, Duchesnea indica (Shemei in Chineses).

### 3.10. Disease target search results

**Figure 8:**
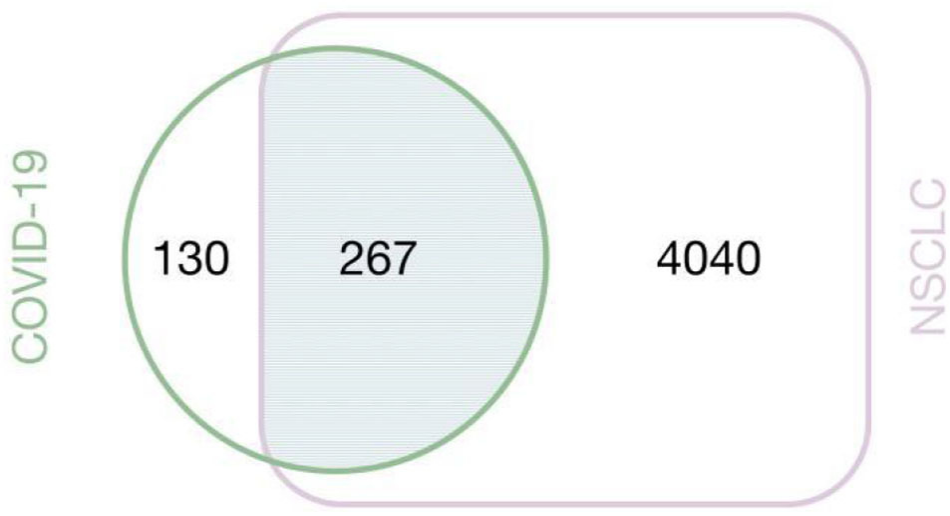
Veen diagram of NSCLC and COVID-19 intersecting targets.

We obtained 397 COVID-19-related targets and 4,307 NSCLC-related targets in total, of which 267 were associated with COVID-19 and NSCLC (Figure 8).

### 3.11. TARGET-INGREDIENT-DRUG of the Zilongjin tablets on the NC

There are 29 targets where the gene targets of the Zilongjin tablets intersect with the NC (Table 3).

**Table 3:** TARGET-INGREDIENT-DRUG of the Zilongjin tablets on the NC.

**TARGET**	**INGREDIENT**	**DRUG**	**TARGET**	**INGREDIENT**	**DRUG**
AKT1	Apigenin, Naringenin, Paeonol, Solamargine, Higenamine, Farnesol Curcumin, Calycosin, Zerumbone, Protocatechuic acid, Salvianolic acid a, Ursolic acid, Albiflorin, Refeldin a, Salvianolic acid b	AR, HSL, SLR	NFKBIA	Resveratrol, Oridonin, Celastrol, Diallyl trisulfide, Ellipticine, Dioscin, Sesamol, Ursolic acid, Wedelolactone, Betanin Butein, Cantharidin	SLR
TNF	Rutin, Scutellarin, Paeoniflorin, Cinnamaldehyde, Farnesol, Curcumin, Salvianolic acid a, Wogonin, Wogonoside, Physostigmine, Ursolic acid, Baicalein, Rhein, Salvianolic acid b	ASR, HSL, SLR	TLR4	Stigmasterol, Cinnamaldehyde, Curcumin, Salvianolic acid b	SLR
BECN1	Curcumin	ASR	STAT3	Apigenin, Lupeol, Cryptotanshinone, Farnesol, Curcumin, Calycosin, Coumarin, Ursolic acid, Capillarisin, Rhein	SLR
EDN1	Resveratrol, Osthole, Emodin	HSL	TGFB1	Apigenin, Cryptotanshinone, Scutellarin, Dihydrotanshinone i, Curcumin, Calycosin, Carnosol	SLR
HIF1A	Dihydrotanshinone i, Curcumin, Salvianolic acid b	HSL	HMOX1	Curcumin, Calycosin, Salvianolic acid a, Baicalein, Salvianolic acid b	SBH, CR
AVP	Acetylcholine	HSL	MPO	Rutin	SBH, CR
MIF		HSL	IL2	Apigenin, Naringenin, Curcumin	SBH
EGFR	Calycosin, Ursolic acid, Rhein	HSL	IFNG	Apigenin, Rutin, Naringenin	SBH
FASN	Curcumin	HSL, CR	IL10	Curcumin, Protocatechuic acid, Salvianolic acid b	SBH
SELP	Curcumin	HSL, CR	F3	Adenosine, Cinnamic acid, Curcumin, Curcumine, Hexanal, Quercetin	SBH
NOS3	Resveratrol, Nuciferine, Diallyl trisulfide, Tauroursodeoxycholic acid, Boldine, Cannabidiol	SLR, SBH, CR	GSTM1	Acetic acid, Curcumin, Curcumine, Kaempferol, Quercetin	CR
NOS2	Neocryptotanshinone, Cryptotanshinone, Curcumin, Salvianolic acid a, Wogonin	SLR	CAT	Apigenin, Scopoletin	CR
IL6	Lupeol, curcumin, Calycosin, Salvianolic acid a, Wogonin Ursolic acid, Brefeldin a, Rhein	SLR, SBH	MMP3	Succinic acid Wogonin	CR
IL1B	Rutin, Paeoniflorin, Cinnamaldehyde, Curcumin, Calycosin, Protocatechuic acid, Wogonin, Baicalein, Rhein	SLR, CR	CD274	Apigenin Curcumin	CR
NFKB1	Apigenin, Naringenin, Neocryptotanshinone, Cryptotanshinone, Solamargine, Paeoniflorin, Cinnamaldehyde, Curcumin, Protocatechuic acid, Salvianolic acid a, wogonin, Wogonoside, Ursolic acid, Baicalein	SLR			

### 3.12. PPI data analysis

**Figure 9:**
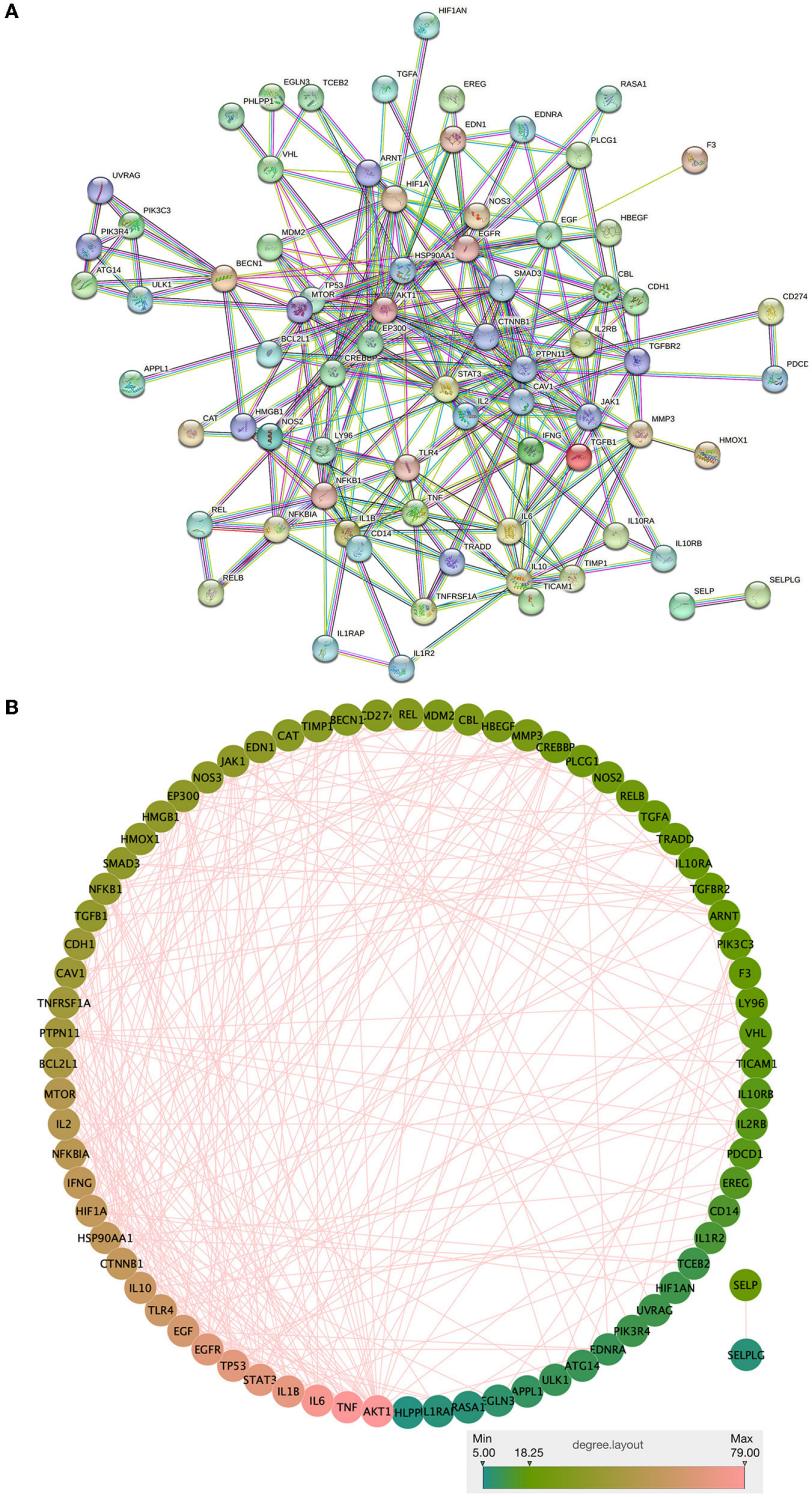
PPI network analysis and visualization of the intersection targets of Zilongjin tablets and the NC.

The intersection targets of drug components and the NC of the Zilongjin tablets were imported into the STRING database, and 74 proteins were obtained (PPI enrichment *p*-value: < 1.0e-16) (Figure 9A). In Cytoscape 3.8.2 software, 74 nodes and 289 edges were obtained using “Analyze network”, sorted by degree value, and the color and size evolved from pink to green with increasing degree value (Figure 9B). TNF (Degree = 79), IL6 (Degree = 76), IL1B (Degree = 70), STAT3 (Degree = 68), TP53 (Degree = 68), EGFR (Degree = 67), EGF (Degree = 62), TLR4 (Degree = 59), IL10 (Degree = 58), HSP90AA1 (Degree = 55), HIF1A (Degree = 55), IFNG (Degree = 54), NFKBIA (Degree = 52), IL2 (Degree = 51), and MTOR (Degree = 50) were the predicted important target proteins.

### 3.13. GO and KEGG enrichment analysis

**Figure 10:**
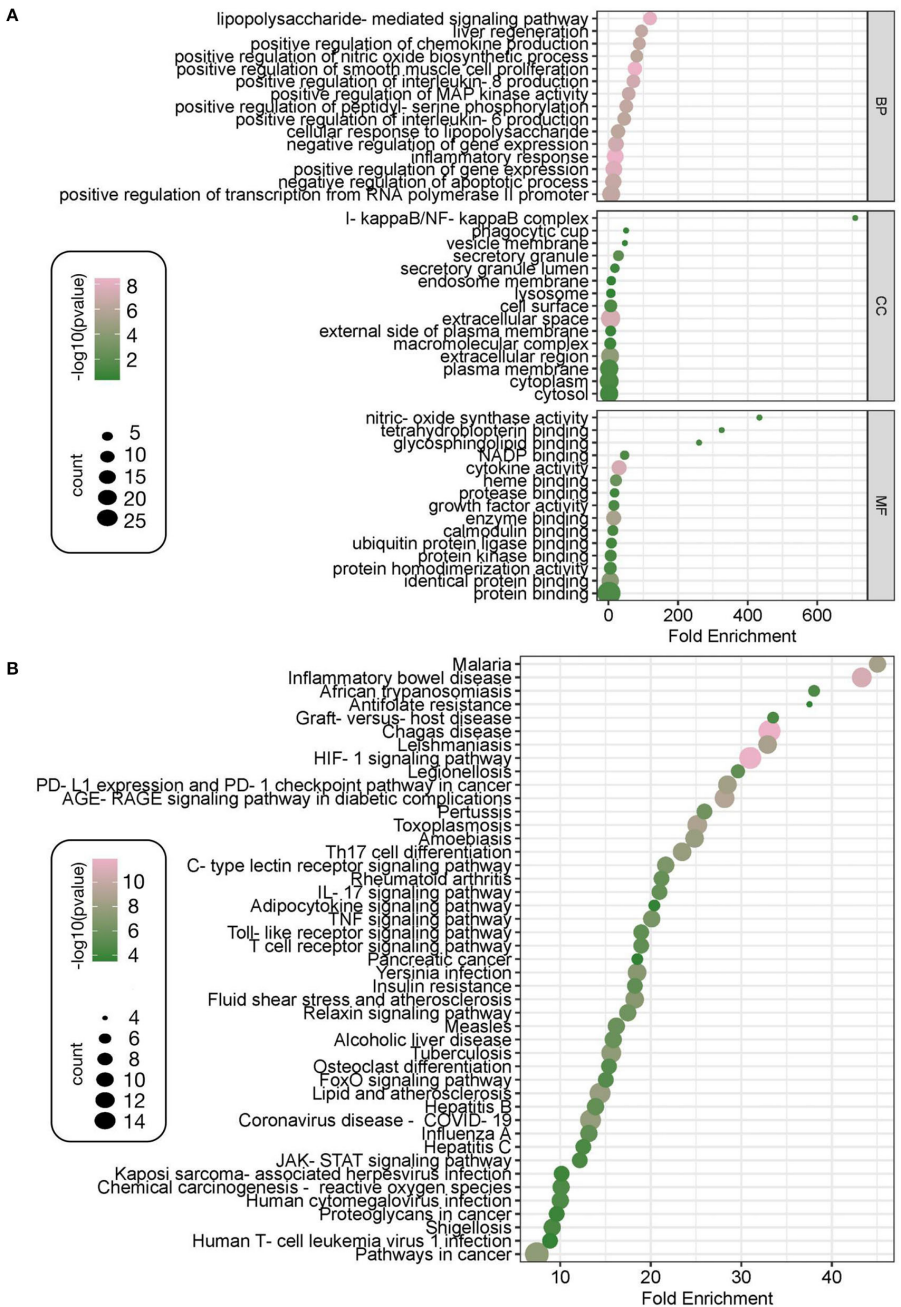
GO and KEGG enrichment analysis. The GO enriched terms of Zilongjin tablets in treating the NC include three parts, the enrichment content of BP (biological process), CC (cellular component), and MF (molecular function), and the 15 results are shown according to the high to low *P* values, respectively. The X-coordinate indicates the fold enrichment, and the color, corresponding to the value of -log10, of the dot represents the *P* value of the terms, the color is pinker to indicate the more significant difference [-log10 (*p*-value)>0.41 means *p* < 0.05 and -log10 (*p*-value) >0.88 means *p* < 0.01]. Besides, the “count” represents the number of genes enriched to this entry, and a bigger dot indicates that more genes are enriched **(A)**. The 45 results of KEGG are shown according to the high to low enrichment of the KEGG pathway **(B)**.

Twenty-nine intersecting genes of the Zilongjin tablets acting on the NC were imported into the David data platform for GO analysis (BP, CC, and MF). A total of 285 entries in total for BP, mainly involving lipopolysaccharide-mediated signaling pathway (*P* < 0.01, count = 7, fold enrichment = 119.50), positive regulation of MAP kinase activity (*P* < 0.01, count = 6, fold enrichment = 58.26) and positive regulation of chemokine production (*P* < 0.01, count = 7, fold enrichment = 88.77), positive regulation of interleukin-8 production (*P* < 0.01, count = 7, fold enrichment = 71.70), et al. (Figure 10A). There were 21 entries for CC, with extracellular space (*P* < 0.01, count = 17, fold enrichment = 6.22), extracellular region (*P* < 0.01, count = 14, fold enrichment = 4.66), and secretory granule (*P* < 0.01, count = 4, fold enrichment = 28.65), et al. related (Figure 10A). MF had a total of 26 entries, including cytokine activity (*P* < 0.01, count = 9, fold enrichment = 30.50), enzyme binding (*P* < 0.01, count = 9, fold enrichment = 15.02), identical protein binding (*P* < 0.01, count = 13, fold enrichment = 4.93), heme binding (*P* < 0.01, count = 5, fold enrichment = 21.54) et al. (Figure 10A). KEGG pathways were enriched with a total of 102 entries, mainly involving Malaria (*p* < 0.01, count = 8, fold enrichment = 45.04), Inflammatory bowel disease (*P* < 0.01, count = 10, fold enrichment = 43.31), African trypanosomiasis (*P* < 0.01, count = 5, fold enrichment = 38.04), Antifolate resistance (*P* < 0.01, count = 4, fold enrichment = 37.54), et al. (Figure 10B).

### 3.14. The network for visual topology analysis of KEGG pathways

**Figure 11:**
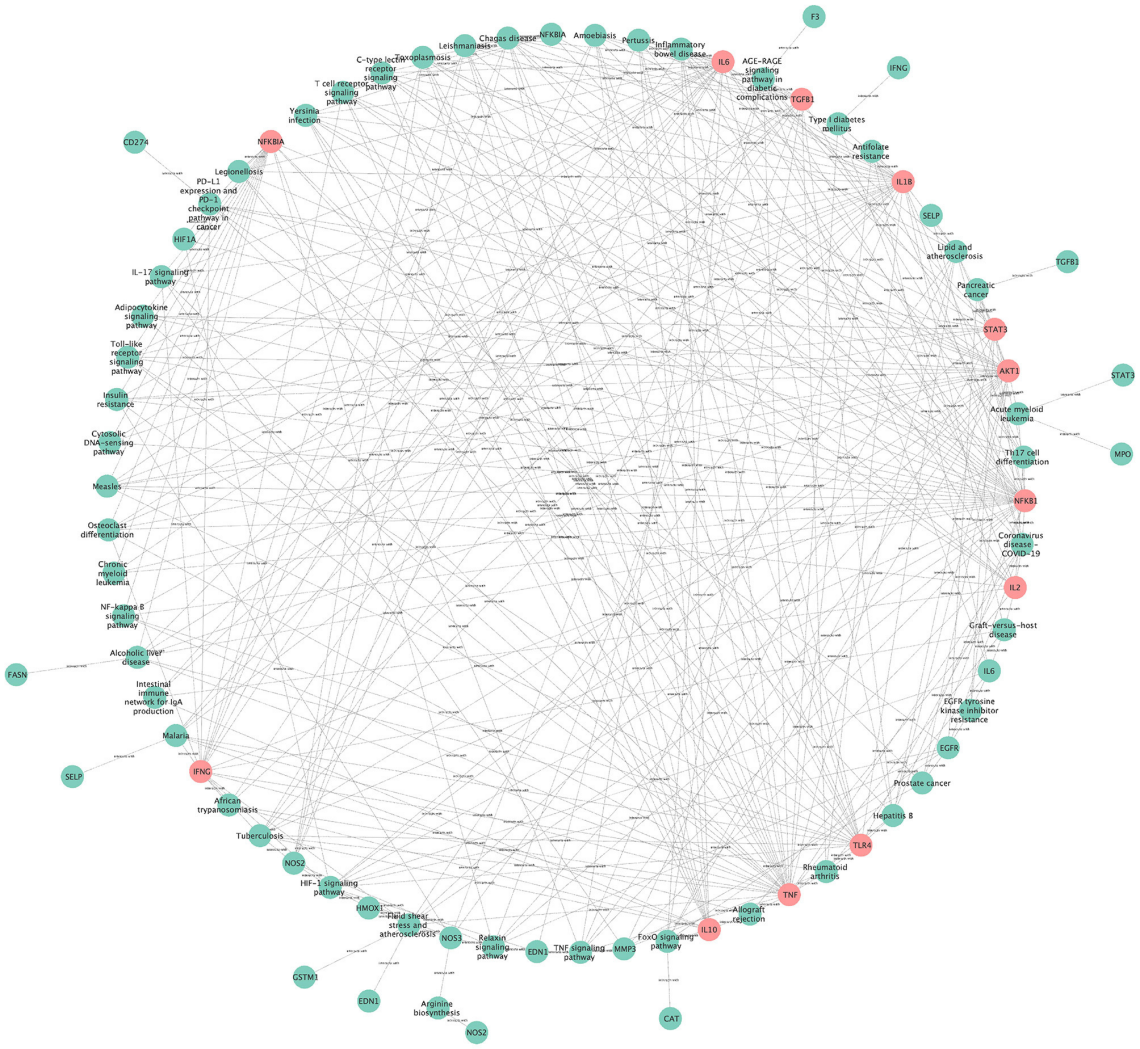
The network for visual topology analysis of KEGG pathways.

The network for visual topology analysis of KEGG pathways contains 79 nodes with 322 edges (Figure 11), the green nodes are pathway genes (Degree < 10), and the pink nodes indicate pathway genes (Degree ≥ 10). The pathways with high degree values include Chagas disease (Degree = 12), Hypoxia-inducible factor 1 (HIF-1) signaling pathway (Degree = 12), Lipid and atherosclerosis (Degree = 11), Coronavirus disease COVID-19 (Degree = 11), Inflammatory bowel disease (Degree = 10), AGE-RAGE signaling pathway in diabetic complications (Degree = 10), Toxoplasmosis (Degree = 10), Tuberculosis (Degree = 10). In addition, targets with Degree values ≥10 include NFKB1 (Degree = 35), TNF (Degree = 31), IL1B (Degree = 28), AKT1 (Degree = 26), IL6 (Degree = 23), TLR4 (Degree = 20), IFNG (Degree = 18), NFKBIA (Degree = 17), TGFB1 (Degree = 16), IL10 (Degree = 15), STAT3 (Degree = 15), and IL2 (Degree = 12).

### 3.15. “Component-target-pathway” network for Zilongjin tables and the NC

**Figure 12:**
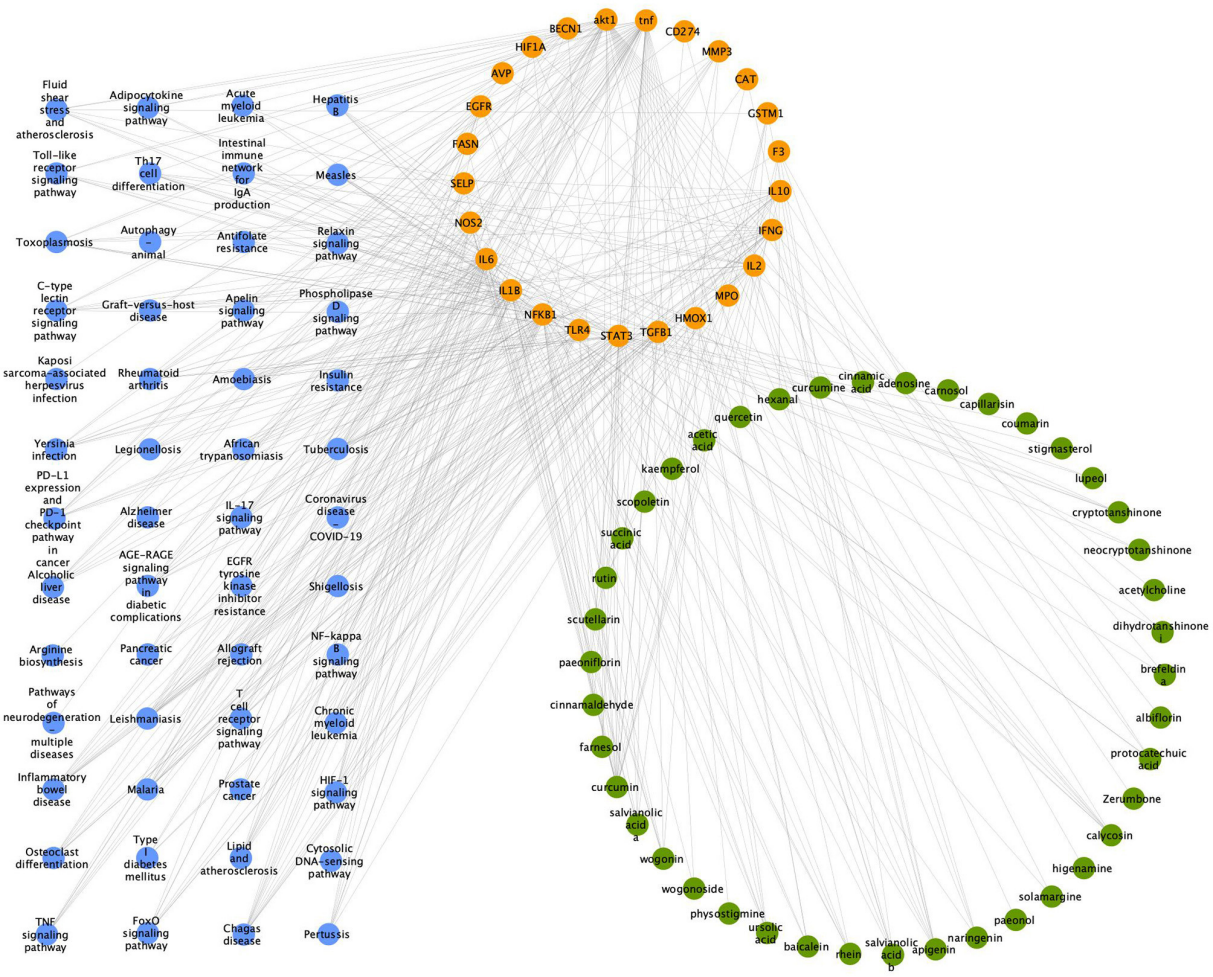
“Component-target-pathway” network for the Zilongjin tablets and the NC. The green nodes represent the drug component, the orange nodes represent the target, and the purple nodes represent the pathway.

The component-target-pathway network consists of 119 nodes and 429 edges. Among them, the 42 green nodes represent the active ingredients of the Zilongjin tablets, with high degree values such as lupeol, curcumin, and apigenin; the 25 pink nodes represent the targets of the Zilongjin tablets on the NC; the 52 purple nodes represent the pathways of the Zilongjin tablets on the NC (Figure 12). Lupeol (degree = 30), curcumin (degree = 19), apigenin (degree = 8), and calycosin (degree = 7) are the main components of the Zilongjin tablets acting on the NC. While Chagas disease (degree = 13), Tuberculosis (degree = 12), HIF-1 signaling pathway (degree = 11), Amoebiasis (degree = 11), and Inflammatory bowel disease [NFKB1 (degree = 48), TNF (degree = 45), AKT1 (degree = 41), IL1B (degree = 37), IL6 (degree = 37), and STAT3 (degree = 36)] are the main pathways through which the active ingredients of the Zilongjin tablets act on the NC. are the main targets of the Zilongjin tablets-the NC.

## 4. Discussion

Lung cancer continues to be the most lethal disease in the world (33). The 5-year survival rate for lung cancer has increased over the past 10 years as a result of improvements in the treatment paradigm (34–39). Given that chemotherapy has considerable toxic side effects, including hematological and digestive side effects, it is imperative to consider combining it with other treatment alternatives to reduce the side effects and improve therapeutic outcomes. Adverse effects like vomiting, constipation, diarrhea, weakness, and poor appetite during treatment leave patients with profound memories of harm and affect their subsequent tolerance and adherence to treatment or even abandonment of treatment. It is true even though potent anti-emetic drugs, leukocyte- and platelet-raising drugs after myelosuppression ensure that chemotherapy is administered on time and in adequate doses. There is a dearth of evidence-based evidence, even though the Zilongjin tablets have been demonstrated to be beneficial in treating lung cancer. Comparing chemotherapy alone to chemotherapy plus the Zilongjin tablets, we found that combination therapy had a better impact on NSCLC outcomes and patients' quality of life and that treatment-induced hematological side effects, digestive system side effects, and hair loss were less severe.

Phlegm buildup and blood stagnation, which prevent the Qi of the lung from spreading and purifying, are the fundamental pathogenic factors for lung cancer. Cancer patients were experiencing immunity declining as the disease and its treatments and were often in touch with medical facilities during the COVID-19 outbreak because they needed therapy. Owing to hypo immunity and the lung damage caused by their lung cancer, patients are more susceptible to the SARS-CoV-2 of the COVID-19 virus, which can further exacerbate their condition. According to some disease reports, COVID-19 can cause severe infections and even death (40, 41). With the mortality rate ranging from 3 to 6%, multi-organ failure, acute respiratory distress syndrome, and septic shock occur in almost 20% of patients with COVID-19 (42). Although several therapies have been tried, none of them succeeded. Clinical care focuses more on symptom management, including organ transplantation in the ICU for patients who are severely sick.

According to the theory of traditional Chinese medcine (TCM), “Yili's qi” is the main cause of COVID-19. Moreover, COVID-19's occurrence and development are affected by several pathogenic variables, including moisture, toxicity, cold, heat, and stasis, and the interpretation of the cause is intricate. Many researchers have dissected the interpretation of the cause of COVID-19 from the perspectives of the “warm and turbid,” “heat-toxin,” “cold-dampness,” “cold-damp, the volatile-dry combo,” and “plague-toxicity”, but the conclusion has not been unified. Nowadays, the majority of studies categorize COVID-19 into four phases: (1) Early-stage symptoms might include cold-dampness invading the lungs, heat-dampness invading the lungs, etc. (2) Middle-late-stage symptoms gradually deepen the degree, “appearing to become heat due to depression” (the pathogenic factor transforms into the heat-toxin due to long-term stagnation), “the stagnation of pathogenic dampness and heat” (the combination of dampness and heat damages the body), “phlegm obstructs the lung” (due to difficulty to remove, the dampness gradually develops into a new pathogenic factor “phlegm-dampness”, which obstructs the function of the lung and causes lung damage), and “syndrome of lung collaterals injured by heat” (because the pathogenic factor “warmth-hotness” is more powerful, it depletes the lung fluid and burns the blood vessels in the lungs, forming blood stasis.). (3) In the recovery period, there is “intermingled deficiency and excess”. Although the healthy qi expelling pathogen, the qi is insufficient and the qi, blood, yin, and yang are deficient. Meanwhile, there is a possibility of recurrence of the disease due to the small amount of pathogen vested in the body. (4) In the critical stage, there are several symptoms, such as “syndrome of epidemic toxin blocking lung” (the pathogenic factor “epidemic toxin” blocking the lung qi, resulting in more serious lung ventilation and systemic symptoms).

Yet, there is no variation in the viral strains' capacity to infect various groups when people are infected with the same type of COVID-19 virus (SARS-CoV-2). The varying immunological capacities of various people are an influencing factor in disease severity. Because of high-level immunity, the general population with COVID-19 often has milder symptoms. In contrast, patients with COVID-19 combined NSCLC generally have more severe symptoms due to low-level immunity, and the disease symptoms are mainly intermingled deficiency and excess. The following explanations apply: (1) NSCLC patients have lung damage, so lung qi is weaker to resist pathogenic factors (The lung controls the Qi of the entire body, and the lung Qi has the function to resist pathogenic factors according to TCM theory). Additionally, the DUXIE (pathogenic factor) invading their bodies may even be guided by the patient's inherent pathogenic factors, aggravating the inherent condition. Then the pathogenic factors directly cause serious damage to the lungs, resulting in symptoms such as “phlegm obstructs the lung”, “syndrome of lung collaterals injured by heat”, and “syndrome of epidemic toxin blocking lung”. (2) NSCLC patients who have undergone chemotherapy have a deficient constitution, an imbalance of qi, blood, yin and yang, and dysfunction of the viscera. They are less able to fend off the virus than healthy individuals, which causes their disease state to change more quickly and unstablely than healthy individuals and increases their risk of the disease.

The primary goal of treatment in the early, middle and late phases of the disease in healthy individuals with COVID-19 is to eradicate the DUXIE (pathogenic factor), avoiding the use of tonic formulations or medications that may induce DUXIE (pathogenic factor) stacking. Patients with COVID-19 and NSCLC, on the other hand, require a mix of corrective (the application of tonics) and detoxifying (the removal of pathogenic elements) treatment.

The Zilongjin tablets, as a TCM treatment for lung cancer, have the ability to benefit Qi and nourish the blood, clear heat and detoxify the blood, and regulate Qi and resolve stasis. In this study, the mechanism of action of the Zilongjin tablet on COVID-19 combined with NSCLC was discussed through network pharmacology. The top three major drug components of the Zilongjin tablets are lupeol, curcumin, and apigenin, as shown in the “Component-Target-Pathway” diagram. Lupeol is a natural product found in the AR and other organisms with data available. It acts through numerous signaling pathways such as Inflammatory bowel disease and HIF-1 signaling pathway, and target IL6, STAT3. Curcumin is a natural dyestuff found in the ASR and CR, and it has a role as an anti-inflammatory agent, an antineoplastic agent, a hepatoprotective agent, and an anti-inflammatory agent. Additionally, antifolate resistance, the HIF-1 signaling pathway, and legionellosis are a few of the many signaling pathways through which it functions, and it targets NOS2, IL6, IL1B, NFKB1, TLR4, STAT3, BECN1, HIF1A, FASN, SELP, and other molecules. A natural substance called apigenin is present in SLR, SBH, and other organisms for which data are available, and functions as a metabolite modulator and antineoplastic agent, targeting NFKB1, STAT3, TGFB1, IL2, IFNG, CAT, CD274, and IFNG. These genes are involved in the inflammatory response, and inflammation-triggered immunoreaction plays a significant role in both NSCLC and COVID-19. Moreover, COVID-19-induced endothelial dysfunction will further exacerbate pathological transformation and cause more pronounced inflammation. A cohort study reported that the Neutrophil-to-lymphocyte ratio and platelet-to-lymphocyte ratio were discovered to be separate predictors of mortality in patients with NSCLC neo-coronary pneumonia (43). The Zilongjin tablets will relieve disease progression and reduce patient death brought on by overly aggressive inflammatory responses when they act on the NC. Additionally, several studies have hypothesized that the increased risk for NC patients may relate to lung damage and the elevated immunosuppression (42). The Zilongjin tablets act on the NC via a bidirectional immunoregulation in which BP includes both positive and negative regulation of the inflammatory response. It can inhibit the excessive proliferation of inflammatory cells such as T cells or inflammatory factors, thus suppressing the excessive inflammatory response and positively regulating the inflammatory response to avoid immunosuppression. The further effect of the Zilongjin tablets on the NC needs to be verified by subsequent experiments.

There are some limitations to this study. Fourteen RCTs were national studies and may be subject to some bias. Three included studies explicitly stated that they used a randomization table to achieve randomization, while other studies did not report randomization procedures, and only 1 article reported a double-blind approach, factors that may contribute to implementation bias, and measurement bias. Although objective response rate analyses reported that the Zilongjin tablets in combination with chemotherapeutic agents were significantly more effective than chemotherapy alone in the treatment of NSCLC. According to objective response rate analyses, due to the small sample sizes of the included studies, there was some clinical heterogeneity that affected the analysis's outcome. The dosage of the Zilongjin tablets was consistent across the RCTs (2.6 g/dose, orally, 3 times/d), but the chemotherapy regimens used varied, with two chemotherapy regimens set up in Wang and Yan study (28) (i) norethindrone (VNB) 25 mg/m^2^ + cisplatin 25 mg/m^2^; (ii) mitomycin (MMC) 6 mg/m^2^ + vincristine (VDS) 3 mg/m^2^ + cisplatin (DDP). The other 10 studies designed only one chemotherapy regimen, such as irinotecan 175 mg/m^2^ + carboplatin (AUC = 5), docetaxel 75 mg/m^2^ + cisplatin 20 mg/m^2^, gemcitabine hydrochloride 1,000 mg/m^2^ + cisplatin 60 mg/m^2^, etc. The chemotherapy regimens were not consistent across studies and may have an impact on treatment outcomes, as well as on the results of this study.

## 5. Conclusion

In this study, we comprehensively evaluated the efficacy of the Zilongjin tablets in the treatment of NSCLC. The efficacy and the degree of improvement in the quality of life of the Zilongjin tablets combined with chemotherapy in the treatment of NSCLC were significantly better than those of chemotherapy alone, and they could attenuate various adverse effects brought about by the disease or chemotherapy. In the network pharmacology, it reported that the efficacy of the Zilongjin tablets against NSCLC combined with COVID-19 might be achieved through the bidirectional regulation of inflammation, while further laboratory analysis was required for subsequent efficacy verification.

## Data availability statement

All data used in this article are from public databases. The original contributions presented in the study are included in the article/supplementary material, further inquiries can be directed to the corresponding author.

## Author contributions

WY and XX contributed to the conception and design of the study. WY, YZ, and YY organized the literature review database and performed the statistical analysis. JG, PH, and XX organized the pharmacological database and performed the statistical analysis. WY, YZ, and XX wrote the first draft of the manuscript. JG wrote sections of the manuscript. JG and XX contributed to the manuscript revision. All authors read and approved the submitted version.
